# 超高效液相色谱-串联质谱法快速测定化妆品中87种禁用原料

**DOI:** 10.3724/SP.J.1123.2023.04012

**Published:** 2024-01-08

**Authors:** Bei HU, Lixia LI, Xiaoping DING, Hong LIU, Wei HUANG, Wen LÜ, Xiaojian LI

**Affiliations:** 湖北省药品监督检验研究院, 湖北省药品质量检测与控制工程技术研究中心, 湖北 武汉 430075; Hubei Institute for Drug Control, Hubei Engineering Research Center for Drug Quality Control, Wuhan 430075, China

**Keywords:** 超高效液相色谱-串联质谱, 禁用原料, 化妆品, ultra performance liquid chromatography-tandem mass spectrometry (UPLC-MS/MS), prohibited ingredients, cosmetics

## Abstract

建立了超高效液相色谱-串联质谱测定化妆品中87种禁用原料的方法,其适用于水基类、乳液膏霜类和油基类3种常见化妆品基质。实验对提取溶剂、提取时间及色谱、质谱条件进行了考察。水基类和乳液膏霜类化妆品用乙腈分散、50%乙腈水溶液超声提取,油基类化妆品用正己烷分散、70%乙腈水溶液涡旋提取;采用CORTECS C_18_色谱柱(150 mm×2.1 mm, 2.7 μm)进行分离,以乙腈和0.1%甲酸水溶液为流动相进行梯度洗脱,流速为0.3 mL/min,柱温为40 ℃,进样量为2 μL。采用电喷雾电离(ESI)源,在多反应监测(MRM)模式下采集数据,利用基质匹配标准曲线进行定量分析。结果表明,87种禁用原料在各自的线性范围内具有良好的线性关系,相关系数(*r*)>0.99,检出限(LOD)为0.07~0.38 μg/g,定量限(LOQ)为0.21~1.15 μg/g。3种化妆品基质中87种禁用原料在低、中、高3个加标水平下的加标回收率为81.7%~115.4%,相对标准偏差(RSD, *n*=6)为0.4%~9.9%。采用该方法对349批化妆品样品进行测定,共发现8批阳性样品,检出成分分别为甲氧苄啶、特比萘芬、萘甲唑啉、7-甲氧基香豆素和7-甲基香豆素。本方法前处理过程简便,定性、定量可靠,可用于测定化妆品中87种禁用原料,能够为化妆品的快速风险筛查和国家标准的制修订提供技术支撑。

## Abstract (en)

The methods of detecting numerous prohibited components are not included in the Technical Specifications for Cosmetic Safety (2015 Edition). Recently, owing to its high speed, sensitivity, and anti-interference properties, ultra performance liquid chromatography-tandem mass spectrometry (UPLC-MS/MS) became the preferred method of detecting banned substances in cosmetics. In this study, a UPLC-MS/MS method was developed for use in determining 87 prohibited ingredients in cosmetics, including 33 sex hormones, 20 anti-infective drugs, 15 antihistamines, 7 coumarins, 4 sedative-hypnotic drugs, 4 antipyretic and analgesic drugs, 2 allergenic fragrances, and 2 drugs with vasoconstriction effects. The main factors affecting the response, recovery, and sensitivity of the method, such as the type of extraction solvent, extraction time, ratio of the mobile phases, and MS conditions, were optimized during sample pretreatment and instrumental analysis. Accordingly, approximately 0.2 g of the toner or cream sample was dispersed in 2 mL acetonitrile in a 10 mL colorimetric tube. After diluting to 10 mL with 50% acetonitrile aqueous solution, the sample was ultrasonically extracted for 20 min and centrifuged, and the mixture was then filtered through a 0.22 μm membrane. Approximately 0.2 g of the oil sample was dispersed in 2 mL *n*-hexane in a 15 mL polypropylene centrifuge tube and extracted twice with 3 mL 70% acetonitrile aqueous solution. The extracts were transferred into a 10 mL colorimetric tube and diluted to 10 mL with 50% acetonitrile aqueous solution, and the mixture was then filtered through a 0.22 μm membrane. The samples were separated using a CORTECS C_18_ column (150 mm×2.1 mm, 2.7 μm), employing a gradient elution program with acetonitrile and 0.1% formic acid aqueous solution as the mobile phases. The flow rate, column temperature, and injection volume were respectively set at 0.3 mL/min, 40 ℃, and 2 μL. The 87 compounds were monitored in multiple reaction monitoring (MRM) mode with electrospray ionization (ESI) under positive and negative conditions. Matrix-matched external standard calibration was used for quantification, and the analysis was completed within 33 min. The prohibited compounds exhibited good linear relationships, with *r* values of >0.99, and the limits of detection (LODs) and quantification (LOQs) for the 87 compounds were 0.07-0.38 and 0.21-1.15 μg/g, respectively. Three types of cosmetic matrices were selected to verify the recovery and precision of the method at LOQ, 2 LOQ, and 10 LOQ levels. The average recoveries of the 87 prohibited compounds were in the range of 81.7%-115.4%, and the relative standard deviations (RSDs, *n*=6) were 0.4%-9.9%. The reliability of the developed method was demonstrated by applying it to 349 commercial cosmetics obtained from the market, and 8 positive samples were identified. The positive components included trimethoprim, terbinafine, naphazoline, 7-methoxycoumarin, and 7-methylcoumarin. The established method displays the advantages of simple operation and rapidness and a high sensitivity and good recovery. And, this method provides technical support for rapid risk screening and the revision of national standards for cosmetics.

《化妆品安全技术规范》(2015年版)^[1]^是国家及省级化妆品监督抽检工作的检验和判定依据,其中列出的禁用原料按是否具有功效分为两类:一类在化妆品中不具有功效,且不太可能以原料或杂质的形式出现在化妆品中;另一类在化妆品中具有一定的功效,如具有美白祛斑、抗菌消炎、抗过敏、改善红血丝、舒缓助眠和增加香味等功能。目前,许多禁用原料在《化妆品安全技术规范》(2015年版)中尚无检测方法,但已有学者检出了非法添加标准方法以外的禁用原料^[2⇓-4]^,因此,化妆品禁用原料的筛查范围亟待扩展,筛查维度也亟需加大。

目前,化妆品中多类别禁用原料的同时检测方法主要有液相色谱-高分辨质谱法^[5⇓-7]^和液相色谱-串联质谱法^[8⇓⇓-11]^。液相色谱-高分辨质谱法可以获得精确的质量数,为目标化合物乃至未知物分析提供全面的数据^[12]^,但仪器价格昂贵,检验成本高,在方法推广上有一定难度。超高效液相色谱-串联质谱法具有特异性好、灵敏度高等优点,能快速、准确地定量化妆品中的禁用原料,在化妆品中性激素类^[13⇓-15]^、抗感染类^[16,17]^、抗组胺类^[18-19]^、镇痛类^[20]^等药物的检测方面均有应用,是目前化妆品禁用原料检测标准方法建立和日常监督检验的首选方法。

本文基于超高效液相色谱-串联质谱,通过优化前处理和仪器条件,建立了化妆品中87种禁用原料的分析方法。87种禁用原料分别为33种性激素类、20种抗感染类、15种抗组胺类、7种香豆素类、4种镇静催眠类、4种解热镇痛类、2种致敏类药物及2种具有收缩血管作用的药物。本方法适用于3类常见化妆品基质(水基类、乳液膏霜类和油基类)的检测,可为化妆品中禁用原料的快速风险筛查和高效日常监管提供技术支持。

## 1 实验部分

### 1.1 仪器、试剂与材料

Acquity UPLC H-CLASS超高效液相色谱仪、Xevo TQ-S质谱仪(美国Waters公司); HMV-50A多管漩涡混合器(上海珂淮仪器有限公司); LC-250超声波清洗器(山东济宁鲁超超声设备有限公司); ST16台式离心机(德国Thermo Fisher Scientific公司); Milli-Q纯水机(美国Millipore公司)。

甲醇和乙腈(色谱纯,德国默克公司);正己烷(分析纯,国药集团化学试剂有限公司);实验用水为GB/T 6682中规定的一级水。87种禁用原料标准品购自中国食品药品检定研究院、德国Dr. Ehrenstorfer公司、坛墨质检标准物质中心、上海义淮生物有限公司、广州佳途科技股份有限公司、上海甄准生物科技有限公司、上海安谱璀世标准技术服务有限公司、上海源叶生物科技有限公司、天津阿尔塔科技有限公司和美国Stanford Chemicals公司,纯度为95.0%~99.9%(CAS号见表1)。

**表1:** 87种禁用原料的CAS号、保留时间和质谱参数

No.	Compound	CAS No.	Retention time/min	Parent ion (m/z)	Daughter ions (m/z)	CV/ V	CEs/ eV	ESI mode
1	ranitidine (雷尼替丁)	66357-59-3	2.67	315.2	176.1^*^, 130.1	45	25, 45	+
2	sulfacetamide (磺胺醋酰)	144-80-9	3.02	215.1	156.0^*^, 92.1	30	15, 30	+
3	dimetridazole (二甲硝咪唑)	551-92-8	3.48	142.0	96.0^*^, 81.0	45	20, 30	+
4	ronidazole (洛硝达唑)	7681-76-7	3.68	201.1	140.1^*^, 55.1	25	15, 30	+
5	sulfadiazine (磺胺嘧啶)	68-35-9	3.93	251.1	92.1^*^, 156.1	40	35, 25	+
6	emedastine (依美斯汀)	87233-62-3	4.76	303.3	246.2^*^, 174.2	60	30, 40	+
7	doxylamine (多西拉敏)	469-21-6	5.48	271.2	182.2^*^, 167.2	30	20, 40	+
8	trimethoprim (甲氧苄啶)	738-70-5	5.58	291.2	123.1^*^, 230.2	60	30, 30	+
9	marbofloxacin (麻保沙星)	115550-35-1	5.61	363.1	72.1^*^, 320.2	50	35, 25	+
10	tinidazole (替硝唑)	19387-91-8	5.63	248.1	121.0^*^, 128.0	45	20, 30	+
11	lomefloxacin (洛美沙星)	98079-52-8	5.99	352.2	265.2^*^, 308.2	50	35, 25	+
12	danofloxacin (达氟沙星)	112398-08-0	6.00	358.2	340.2^*^, 96.1	55	35, 40	+
13	methoxyphenamine (甲氧那明)	5588-10-3	6.01	180.2	121.1^*^, 149.1	35	25, 15	+
14	sulfamethazine (磺胺二甲嘧啶)	57-68-1	6.09	279.1	186.1^*^, 156.0	40	25, 25	+
15	orbifloxacin (奥比沙星)	113617-63-3	6.24	396.1	352.2^*^, 295.2	50	25, 35	+
16	naphazoline (萘甲唑啉)	550-99-2	6.27	211.3	141.1^*^, 115.1	35	28, 37	+
17	ornidazole (奥硝唑)	16773-42-5	6.28	220.1	128.1^*^, 82.1	45	20, 40	+
18	gatifloxacin (加替沙星)	112811-59-3	6.41	376.2	261.2^*^, 332.2	50	40, 25	+
19	sparfloxacin (司帕沙星)	110871-86-8	6.70	393.2	349.2^*^, 292.2	60	30, 40	+
20	methapyrilene (美沙吡林)	135-23-9	6.88	262.2	97.1^*^, 217.1	35	35, 20	+
21	cinoxacin (西诺沙星)	28657-80-9	7.61	263.1	245.1^*^, 189.1	40	20, 40	+
22	triprolidine (曲普利啶)	6138-79-0	8.13	279.2	208.2^*^, 193.2	30	20, 45	+
23	bromisoval (溴米索伐)	496-67-3	8.27	223.5	179.9^*^, 57.1	30	10, 18	+
24	xanthotol (8-羟基补骨脂素)	2009-24-7	8.33	203.1	147.1^*^, 131.1	50	30, 30	+
25	ketotifen (酮替芬)	34580-14-8	8.34	310.1	96.1^*^, 97.1	60	40, 50	+
26	phenacetin (非那西丁)	62-44-2	8.38	180.1	110.2^*^, 138.1	30	18, 15	+
27	oxolinic (恶喹酸)	14698-29-4	8.51	262.1	244.1^*^, 216.1	30	25, 40	+
28	mizolastine (咪唑斯汀)	108612-45-9	8.78	433.3	109.1^*^, 308.3	50	55, 35	+
29	olopatadine (奥洛他定)	140462-76-6	9.77	338.3	247.2^*^, 165.1	50	30, 35	+
30	decloxizine (去氯羟嗪)	13073-96-6	10.09	341.2	167.1^*^, 152.2	40	25, 60	+
31	chlomezanone (氯美扎酮)	80-77-3	10.24	274.2	154.2^*^, 209.2	30	15, 12	+
32	7-methoxycoumarin (7-甲氧基香豆素)	531-59-9	10.28	177.1	121.1^*^, 133.1	50	30, 25	+
33	carbromal (卡溴脲)	77-65-6	10.87	237.0	194.1^*^, 114.2	35	12, 15	+
34	acrivastine (阿伐斯汀)	87848-99-5	11.17	349.2	278.2^*^, 232.3	50	25, 50	+
35	erythromycin (红霉素)	114-07-8	11.20	734.5	158.2^*^, 116.2	45	50, 60	+
36	haloperidol (氟哌啶醇)	52-86-8	11.21	376.9	123.1^*^, 164.9	25	40, 34	+
37	nalidixic acid (萘啶酸)	389-08-2	11.22	233.1	215.1^*^, 187.1	30	20, 35	+
38	xylometazoline (赛洛唑啉)	1218-35-5	11.43	245.1	145.1^*^, 229.2	35	38, 36	+
39	diphenylpyraline (二苯拉林)	147-20-6	11.56	282.2	167.1^*^, 116.2	50	35, 45	+
40	7-methylcoumarin (7-甲基香豆素)	2445-83-2	11.82	161.1	105.1^*^, 117.1	50	25, 20	+
41	flumequine (氟甲喹)	42835-25-6	12.03	262.1	244.2^*^, 202.1	40	25, 45	+
42	propyphenazone (异丙安替比林)	479-92-5	12.66	231.0	189.0^*^, 201.1	30	20, 22	+
43	fluoxymesterone (氟甲睾酮)	76-43-7	13.21	337.3	281.1^*^, 241.2	30	20, 23	+
44	mifepristone (米非司酮)	84371-65-3	13.23	430.4	134.1^*^, 121.6	62	30, 20	+
45	adrenosterone (肾上腺甾酮)	382-45-6	13.33	301.3	257.3^*^, 121.2	40	21, 26	+
46	fexofenadine (非索非那定)	153439-40-8	13.60	502.3	171.2^*^, 466.4	60	50, 40	+
47	trenbolone (群勃龙)	10161-33-8	13.72	271.2	253.2^*^, 199.1	44	19, 22	+
48	chlorphenoxamine (氯苯沙明)	77-38-3	14.05	304.2	215.1^*^, 90.1	30	20, 10	+
49	boldenone (勃地酮)	846-48-0	14.56	287.2	135.2^*^, 269.1	35	18, 12	+
50	nandrolone (诺龙)	434-22-0	15.02	275.2	109.1^*^, 257.2	30	27, 14	+
51	androstadienedione (1,4-雄烯二酮)	897-06-3	15.76	285.2	121.1^*^, 147.2	4	22, 12	+
52	7-ethoxy-4-methylcoumarin (7-乙氧基-4-甲基香豆素)	87-05-8	15.85	205.1	177.1^*^, 121.1	50	25, 35	+
53	methandrostenolone (美雄酮)	72-63-9	15.91	301.2	121.2^*^, 149.2	30	25, 13	+
54	stanozolol (司坦唑醇)	10418-03-8	16.20	329.2	81.2^*^, 95.1	82	42, 38	+
55	terbinafine (特比萘芬)	78628-80-5	16.33	292.2	141.1^*^, 93.1	40	25, 25	+
56	norethisterone (炔诺酮)	68-22-4	17.26	299.1	109.1^*^, 91.1	40	25, 38	+
57	flugestone acetate (氟孕酮醋酸酯)	2529-45-5	17.61	407.3	225.2^*^, 285.2	72	24, 20	+
58	21-hydroxyprogesterone (21-羟基孕酮)	64-85-7	18.09	331.2	108.8^*^, 97.0	45	25, 30	+
59	androstenedione (雄烯二酮)	63-05-8	18.78	287.2	97.1^*^, 109.2	35	21, 24	+
60	ethisterone (炔孕酮)	434-03-7	19.22	313.3	109.2^*^, 97.1	30	25, 21	+
61	epitestosterone (表睾酮)	481-30-1	19.39	289.1	109.1^*^, 97.1	48	26, 20	+
62	17-α-hydroxyprogesterone (17-α-羟基孕酮)	68-96-2	19.42	331.5	109.2^*^, 97.1	30	26, 24	+
63	acenocoumarol (醋硝香豆素)	152-72-7	19.49	354.1	163.1^*^, 296.1	50	25, 25	+
64	drospirenone (屈螺酮)	67392-87-4	20.03	367.3	97.1^*^, 105.2	45	23, 38	+
65	altrenogest (四烯雌酮)	850-52-2	20.28	311.2	227.2^*^, 269.2	50	24, 13	+
66	megestrol (甲地孕酮)	3562-63-8	20.52	343.3	325.1^*^, 187.1	56	16, 20	+
67	clostebol (氯司替勃)	1093-58-9	20.60	323.3	143.1^*^, 131.1	38	25, 25	+
68	medroxyprogesterone (甲羟孕酮)	520-85-4	21.19	345.2	123.2^*^, 97.0	30	24, 25	+
69	dydrogesterone (去氢孕酮)	152-62-5	21.47	313.3	295.2^*^, 173.1	20	12, 23	+
70	17-α-hydroxyprogesterone 17-acetate (17-α-羟基孕酮-17-醋酸酯)	302-23-8	21.77	373.3	313.3^*^, 271.2	52	12, 16	+
71	ebastine (依巴斯汀)	90729-43-4	22.03	470.3	167.2^*^, 203.2	60	45, 45	+
72	trenbolone-acetate (醋酸群勃龙)	10161-34-9	22.17	313.3	253.2^*^, 107.1	15	19, 31	+
73	phenylbutazone (保泰松)	50-33-9	22.28	309.1	120.1^*^, 92.1	30	28, 36	+
74	isoimperatorin (异欧前胡素)	482-45-1	22.54	271.1	203.1^*^, 69.1	30	15, 30	+
75	norethisterone acetate (炔诺酮醋酸盐)	51-98-9	22.66	341.3	281.2^*^, 109.1	35	15, 28	+
76	danazol (达那唑)	17230-88-5	22.70	338.4	148.2^*^, 120.3	30	23, 30	+
77	pyranocumarin (环香豆素)	518-20-7	23.33	323.2	251.1^*^, 73.0	45	25, 25	+
78	testosterone-17-acetate (17-乙酸睾丸激素)	1045-69-8	23.79	331.5	109.1^*^, 97.2	20	27, 21	+
79	19-nortestosterone 17-propionate (丙酸诺龙)	7207-92-3	24.55	331.4	257.2^*^, 109.1	48	14, 28	+
80	clostebol acetate (醋酸氯睾酮)	855-19-6	24.89	365.2	305.2^*^, 143.1	42	11, 25	+
81	testosterone propionate (丙酸睾酮)	57-85-2	25.15	345.2	97.1^*^, 109.2	38	22, 25	+
82	nandrolone phenylpropionate (苯丙酸诺龙)	62-90-8	26.44	407.2	105.2^*^, 257.1	35	26, 16	+
83	atranol (苔黑醛)	526-37-4	9.96	151.0	123.1^*^, 81.0	10	15, 13	-
84	chlorzoxazone (氯唑沙宗)	95-25-0	10.01	168.0	132.1^*^, 76.1	50	25, 30	-
85	chloroatranol (氯化苔黑醛)	57074-21-2	13.80	185.0	157.0^*^, 93.1	35	13, 19	-
86	dienestrol (双烯雌酚)	84-17-3	20.62	265.2	93.0^*^, 249.0	50	25, 24	-
87	hexestrol (己烷雌酚)	84-16-2	20.73	269.2	119.0^*^, 133.0	37	37, 15	-

CV: cone voltage; CE: collision energy; ESI: electrospray ionization; * quantitative ion; +: positive ion mode; -: negative ion mode.

### 1.2 标准溶液的配制

#### 1.2.1 标准储备溶液

分别准确称取10 mg标准品(精确至0.01 mg)置于10 mL容量瓶中,加入乙腈溶解(其中在乙腈中溶解较差的洛美沙星、司帕沙星、氟甲喹、麻保沙星、达氟沙星和加替沙星可以加入少量甲酸促进溶解),定容至刻度并摇匀,配制成质量浓度为1000 mg/L的标准储备溶液,于-20 ℃下冷冻保存。

#### 1.2.2 单标溶液

使用时准确移取20 μL各标准储备溶液,分别置于不同的10 mL容量瓶中,用乙腈稀释定容并摇匀,配制成质量浓度为2 mg/L的各单标中间溶液。再准确移取1 mL各单标中间溶液分别置于不同的10 mL容量瓶中,用乙腈稀释定容并摇匀,配制成质量浓度为200 μg/L的各单标溶液。

#### 1.2.3 混合标准溶液及基质匹配标准溶液

使用时准确移取0.1 mL各标准储备溶液,置于10 mL容量瓶中,用乙腈稀释定容并摇匀,配制成质量浓度为10 mg/L的混合标准中间溶液;再准确移取1 mL混合标准中间溶液,置于10 mL容量瓶中,用乙腈稀释定容并摇匀,配制成质量浓度为1 mg/L的混合标准溶液。临用前,用空白基质提取液稀释混合标准溶液,配制成所需质量浓度的基质匹配标准溶液。

### 1.3 样品前处理

水基类和乳液膏霜类:称取0.2 g样品(精确至0.0001 g)置于10 mL具塞比色管中,加入2 mL乙腈涡旋分散均匀后,再加入50%乙腈水溶液至近刻度,超声提取20 min,静置至室温;之后用50%乙腈水溶液定容至刻度,摇匀,以8000 r/min离心5 min,取上清液过0.22 μm有机滤膜,滤液作为供试品溶液备用。

油基类:称取0.2 g样品(精确至0.000 1 g)置于15 mL具塞离心管中,加入2 mL正己烷涡旋分散均匀后,再加入3 mL 70%乙腈水溶液涡旋2 min,以8 000 r/min离心5 min;用胶头滴管移取下层溶液至10 mL具塞比色管中,再在上层正己烷层加入3 mL 70%乙腈水溶液涡旋2 min,以8 000 r/min离心5 min,用胶头滴管移取下层溶液至同一10 mL具塞比色管中,加入50%乙腈水溶液定容至刻度,摇匀,过0.22 μm有机滤膜,滤液作为供试品溶液备用。

### 1.4 仪器条件

#### 1.4.1 色谱条件

色谱柱:CORTECS C_18_色谱柱(150 mm×2.1 mm, 2.7 μm);流动相:A为乙腈,B为0.1%甲酸水溶液;柱温:40 ℃;流速:0.3 mL/min;进样体积:2 μL。梯度洗脱程序:0~2 min, 5%A; 2~4 min, 5%A~20%A; 4~13 min, 20%A~35%A; 13~16 min, 35%A; 16~18 min, 35%A~45%A; 18~26 min, 45%A~90%A; 26~31 min, 90%A; 31~31.1 min, 90%A~5%A; 31.1~33 min, 5%A。

#### 1.4.2 质谱条件

离子源:电喷雾电离(ESI)源,正、负离子扫描;检测方式:多反应监测(MRM)模式;毛细管电压:3.2 kV(正离子)和3.0 kV(负离子);脱溶剂气流速:900 L/h;脱溶剂气温度:450 ℃;锥孔气流速:150 L/h。其他质谱参数见表1。

## 2 结果与讨论

### 2.1 UPLC-MS/MS条件优化

#### 2.1.1 质谱条件的优化

采用电喷雾电离,分别将87种禁用原料的单标溶液(200 μg/L)注入离子源,在正、负离子扫描模式下对目标化合物进行一级质谱全扫描。正离子模式下,目标化合物均产生[M+H]^+^准分子离子峰;负离子模式下,目标化合物均产生[M-H]^-^准分子离子峰。确定准分子离子峰后对锥孔电压(CV)进行优化,再进行二级质谱扫描,选取丰度较高且干扰较小的2个子离子分别作为定性、定量离子,其中响应较高的离子作为定量离子。再分别优化2个子离子的碰撞能量(CE),经优化后得到的质谱参数见表1。

#### 2.1.2 色谱条件的优化

根据相关文献^[5,6,8⇓⇓-11]^及国家标准^[1]^,同时分离激素类、抗感染类、抗组胺类和解热镇痛类药物等多个类别禁用原料的流动相体系中,水相多采用含有不同体积分数的甲酸水溶液。本研究考察了0.1%甲酸水溶液和甲醇、0.1%甲酸水溶液和乙腈、0.1%甲酸水溶液和0.1%甲酸乙腈3种流动相体系对待测物峰形及响应的影响。结果显示,在流动相体系为0.1%甲酸水溶液和乙腈的条件下,87种目标物的响应最好,且87种禁用原料中母离子和子离子质荷比相同或相近的组分均可得到分离,87种禁用原料的总离子流色谱图见图1。

**图1:**
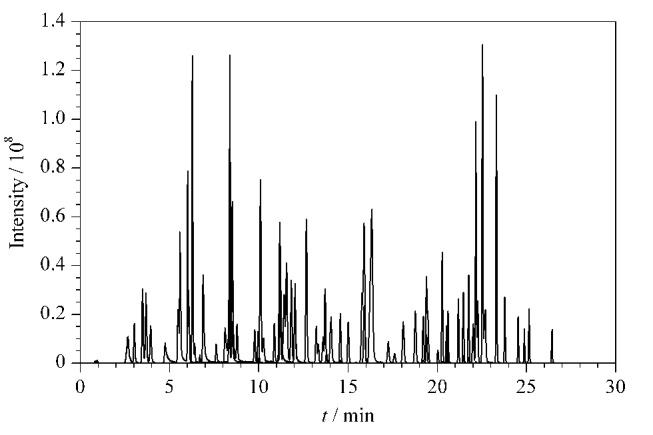
87种禁用原料混合标准溶液的总离子流色谱图

### 2.2 样品前处理条件的优化

#### 2.2.1 水基类、乳液膏霜类样品

提取溶剂的选择 本研究所涉及的87种禁用原料中,大部分禁用原料在乙腈中有较好的溶解性。经查阅相关文献^[13⇓-15,17,20⇓⇓⇓⇓-25]^,本研究以乳液膏霜类样品为典型基质,先加入适量1 mg/L的87种禁用原料混合标准溶液(加标水平均为2倍定量限(LOQ)),之后分别考察了用乙腈、50%乙腈水溶液、70%乙腈水溶液提取及先用乙腈提取再加水定容的4种提取方式对目标物提取效率的影响。结果显示,使用乙腈和70%乙腈水溶液提取时,由于溶剂效应,部分出峰时间较早的化合物色谱峰变宽,峰形对称性差。比较了用50%乙腈水溶液提取和先用乙腈提取再加水定容两种提取方式下目标物的加标回收率,结果如图2所示。在两种提取方式下,87种禁用原料的加标回收率分别为91.7%~116.2%和81.8%~115.7%,相对标准偏差分别为0.7%~8.4%和1.2%~8.2%。使用50%乙腈水溶液进行提取时不仅回收率更高,且操作简便、快捷,更符合化妆品快速筛查的目的,因此,选择50%乙腈水溶液作为水基类和乳液膏霜类样品的提取溶剂。

**图2:**
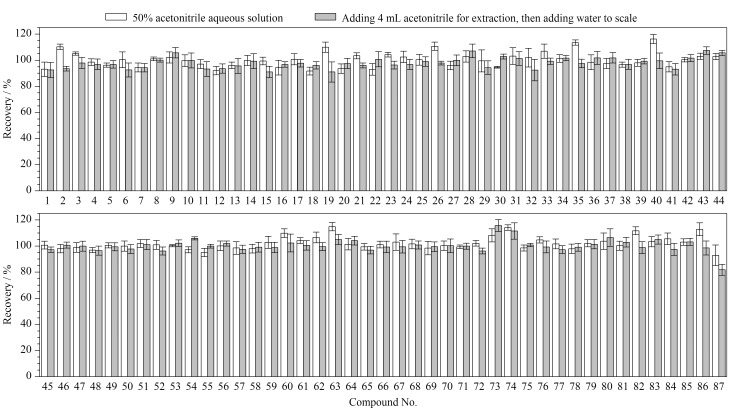
乳液膏霜类基质中87种禁用原料在不同提取溶剂下的加标回收率(*n*=6)

提取时间的选择 以乳液膏霜类样品为典型基质,考察了不同超声提取时间(10、20、30 min)对87种禁用原料提取效率的影响。结果表明,以50%乙腈水溶液为提取溶剂、超声提取20 min的条件下,绝大多数化合物可达最大提取效率。因此,实验选择超声提取时间为20 min。

#### 2.2.2 油基类样品

提取溶剂的选择 根据文献[13,14]报道,本研究在空白油基类化妆品中先加入适量1 mg/L的87种禁用原料混合标准溶液(加标水平均为2倍LOQ),之后分别考察了不同体积分数(50%、70%、80%)的乙腈水溶液作为提取溶剂时对87种禁用原料提取效率的影响。结果如图3所示,使用50%乙腈水溶液提取时,丙酸睾酮和苯丙酸诺龙的加标回收率仅为49.6%和42.4%;使用70%和80%乙腈水溶液提取时,87种禁用原料的加标回收率分别为83.6%~114.9%和78.3%~115.0%,相对标准偏差分别为1.4%~9.1%和0.8%~8.0%。由此可见,70%乙腈水溶液的提取效率更高,且在该提取溶剂下出峰时间较早的化合物的峰形对称性更好。因此,选择70%乙腈水溶液作为油基类样品的提取溶剂。

**图3:**
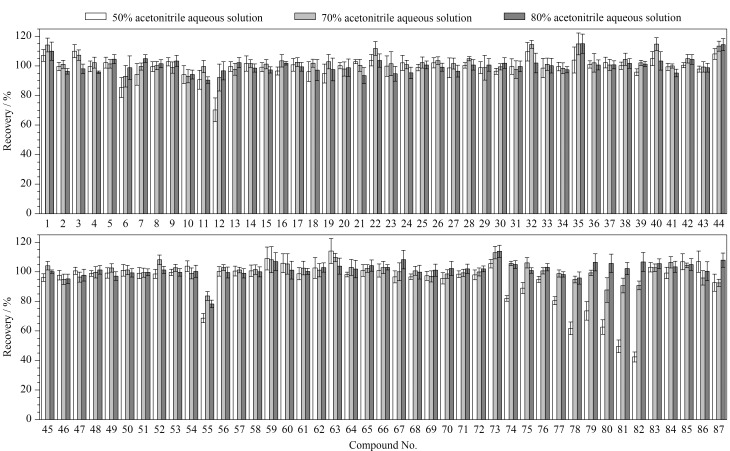
油基类基质中87种禁用原料在不同提取溶剂下的加标回收率(*n*=6)

提取次数的选择 实验分别考察了涡旋提取1、2、3次对87种禁用原料提取效率的影响。仅提取1次时,依美斯汀、丙酸诺龙、醋酸氯睾酮、丙酸睾酮和苯丙酸诺龙5个组分的回收率均小于80%;分别提取2次和3次时,87种禁用原料的加标回收率为83.7%~115.8%和82.0%~112.1%,相对标准偏差分别为0.9%~8.6%和0.6%~8.0%,由此可见,提取2次时已经能够满足87种禁用原料筛查测定的要求。因此,为节省检测时间,选择涡旋提取次数为2次。

### 2.3 基质效应的考察

实验分别考察了3类化妆品基质的基质效应(matrix effect, ME),并采用ME=基质匹配标准曲线斜率/溶剂标准曲线斜率的计算方法对目标化合物的基质效应进行评价;其中,ME>1表现为基质增强效应,ME<1表现为基质抑制效应;ME值越接近1,表示基质效应的影响越小,反之,则表明基质效应的影响越大^[26]^。实验结果表明,3类化妆品基质经溶剂提取后均有部分共萃取基质被提取,水基类、乳液膏霜类和油基类基质的ME分别为0.58~1.82、0.68~7.15和0.89~1.55(图4),均存在较明显的基质抑制或基质增强效应。因此,为了降低基质效应带来的影响,本实验采用基质匹配标准溶液进行定量分析。

**图4:**
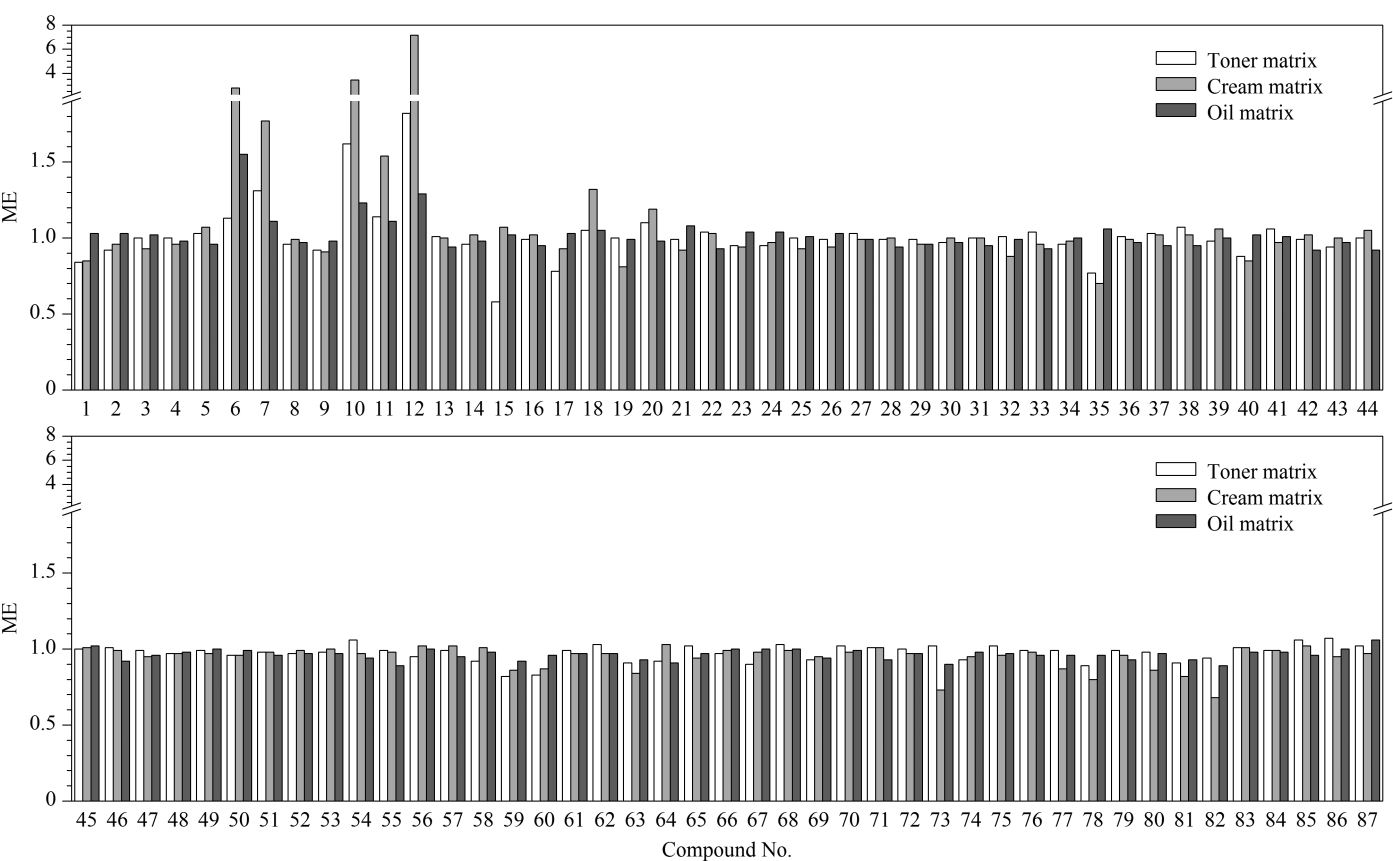
水基类、乳液膏霜类和油基类样品中87种禁用原料的基质效应

### 2.4 线性关系、检出限及定量限

本实验利用空白基质(水基类、乳液膏霜类和油基类基质)提取液对87种禁用原料的混合标准溶液进行稀释,配制成系列质量浓度的基质匹配标准溶液。以待测物的质量浓度(*x*, μg/L)为横坐标、定量离子对的峰面积(*y*)为纵坐标,绘制基质匹配标准工作曲线。结果显示,3种化妆品基质中目标化合物在各自的线性范围内均具有良好的线性关系,相关系数(*r*)均大于0.99。以信噪比≥3计算检出限(LOD),以信噪比≥10计算LOQ, 87种禁用原料的LOD为0.07~0.38 μg/g,LOQ为0.21~1.15 μg/g。3种化妆品基质中87种禁用原料的线性范围、线性方程、相关系数、LOD和LOQ见表2。

**表2:** 3种化妆品基质中87种禁用原料的线性范围、LOD、LOQ、线性方程和相关系数

Compound		Linear range/ (μg/L)	LOD/ (μg/g)	LOQ/ (μg/g)		Toner matrix				Cream matrix					Oil matrix	
						Linear equation		*r*		Linear equation			*r*		Linear equation	*r*
Ranitidine		15.25-228.79	0.31	0.92		*y*=2400.5*x*-1496.4		0.9989		*y*=2371.8*x*-10514.9		0.9993			*y*=2358.5*x*-10101.2	0.9994
Sulfacetamide		16.80-252.02	0.34	1.01		*y*=5655.2*x*+16422.6		0.9992		*y*=5010.9*x*+25181.9		0.9984			*y*=5470.7*x*+25042.9	0.9986
Dimetridazole		15.98-239.66	0.32	0.96		*y*=15697.5*x*-34887.1		0.9997		*y*=12726.0*x*+34942.1		0.9995			*y*=14481.4*x*+4299.7	0.9993
Ronidazole		16.41-246.16	0.33	0.98		*y*=13107.9*x*+6208.7		0.9999		*y*=12348.8*x*+60793.4		0.9984			*y*=12810.3*x*+62525.9	0.9989
Sulfadiazine		16.06-240.93	0.32	0.96		*y*=6018.8*x*+2753.8		0.9988		*y*=6866.5*x*+28266.4		0.9992			*y*=6244.0*x*+27850.5	0.9981
Emedastine		15.47-232.12	0.31	0.93		*y*=3170.6*x*+51874.7		0.9963		*y*=5620.6*x*-7541.9		0.9976			*y*=3177.8*x*+48455.1	0.9966
Doxylamine		4.51-67.66	0.09	0.27		*y*=68123.4*x*-11920.7		0.9996		*y*=75952.9*x*-112280.0		0.9996			*y*=63840.7*x*+11006.1	0.9994
Trimethoprim		5.54-83.16	0.11	0.33		*y*=26457.0*x*+35841.3		0.9985		*y*=26170.3*x*+27613.1		0.9991			*y*=27473.0*x*+33170.0	0.9983
Marbofloxacin		15.66-234.92	0.31	0.94		*y*=1848.5*x*+9219.7		0.9992		*y*=2308.9*x*+1108.7		0.9992			*y*=1423.4*x*+18920.6	0.9978
Tinidazole		4.59-68.87	0.09	0.28		*y*=11224.3*x*+23945.1		0.9969		*y*=11271.9*x*+25361.7		0.9982			*y*=12047.2*x*+17744.2	0.9986
Lomefloxacin		3.99-59.81	0.08	0.24		*y*=5811.0*x*+6712.0		0.9983		*y*=5836.8*x*+2574.9		0.9984			*y*=5393.4*x*+6501.9	0.9989
Danofloxacin		18.57-278.55	0.37	1.11		*y*=3534.7*x*+43071.5		0.9964		*y*=5806.4*x*+1135.3		0.9963			*y*=2419.9*x*+57441.7	0.9963
Methoxyphenamine		4.10-61.52	0.08	0.25		*y*=44721.5*x*+30419.8		0.9988		*y*=42119.0*x*-5667.9		0.9997			*y*=41860.9*x*+48782.6	0.9982
Sulfamethazine		5.07-76.12	0.10	0.30		*y*=11464.0*x*+15139.4		0.9967		*y*=12296.6*x*+31394.0		0.9972			*y*=12280.0*x*+19579.1	0.9995
Orbifloxacin		5.54-83.14	0.11	0.33		*y*=4629.2*x*-155.1		0.9992		*y*=7836.9*x*+7377.6		0.9980			*y*=7760.0*x*+6785.2	0.9991
Naphazoline		5.26-78.91	0.11	0.32		*y*=79884.8*x*+13503.5		0.9988		*y*=82114.5*x*-6091.3		0.9994			*y*=80275.8*x*+100938.0	0.9989
Ornidazole		5.02-75.37	0.10	0.30		*y*=18999.4*x*+7662.7		0.9986		*y*=20769.3*x*+30261.8		0.9997			*y*=23909.7*x*+9342.8	0.9994
Gatifloxacin		5.64-84.66	0.11	0.34		*y*=5899.3*x*+984.0		0.9998		*y*=7174.3*x*-6411.5		0.9993			*y*=6526.4*x*+2898.8	0.9993
Sparfloxacin		5.51-82.71	0.11	0.33		*y*=2657.9*x*+1094.8		0.9991		*y*=2198.2*x*+536.8		0.9987			*y*=2537.4*x*-1482.1	0.9990
Methapyrilene		5.65-84.70	0.11	0.34		*y*=59277.8*x*-21777.6		0.9996		*y*=60609.8*x*-88707.0		0.9997			*y*=56854.6*x*-9936.5	0.9998
Cinoxacin		5.45-81.82	0.11	0.33		*y*=28237.6*x*+30786.1		0.9980		*y*=29013.5*x*+23560.8		0.9991			*y*=30206.0*x*+6551.5	0.9976
Triprolidine		3.55-53.32	0.07	0.21		*y*=127241.0*x*-153180.0		0.9969		*y*=125875.0*x*-133665.0		0.9986			*y*=124424.0*x*-41976.7	0.9998
Bromisoval		14.54-218.10	0.29	0.87		*y*=1389.4*x*-22.3		0.9982		*y*=1256.0*x*+3161.5		0.9990			*y*=1338.4*x*-222.0	0.9994
Xanthotol		14.38-215.65	0.29	0.86		*y*=6562.7*x*+19098.0		0.9984		*y*=6431.5*x*+15426.4		0.9987			*y*=6740.5*x*+4341.0	0.9990
Ketotifen		13.75-206.24	0.27	0.82		*y*=12863.0*x*+40377.9		0.9992		*y*=11579.5*x*+24740.0		0.9991			*y*=12917.4*x*+51614.2	0.9990
Phenacetin		6.32-94.86	0.13	0.38		*y*=65253.3*x*+222964.0		0.9987		*y*=62330.3*x*+270544.0		0.9960			*y*=68191.9*x*+204305.0	0.9987
Oxolinic		5.14-77.10	0.10	0.31		*y*=158433.0*x*+188163.0		0.9994		*y*=151001.0*x*+270534.0		0.9979			*y*=152449.0*x*+242155.0	0.9974
Mizolastine		16.26-243.86	0.33	0.98		*y*=6281.3*x*+30442.4		0.9984		*y*=6307.9*x*+29549.3		0.9978			*y*=5662.5*x*+40439.6	0.9985
Olopatadine		3.98-59.77	0.08	0.24		*y*=15869.8*x*-920.2		0.9995		*y*=15352.8*x*+9094.2		0.9985			*y*=14947.1*x*+2584.8	0.9997
Decloxizine		3.85-57.68	0.08	0.23		*y*=142884.0*x*+13327.1		0.9994		*y*=140014.0*x*-63284.8		0.9996			*y*=143375.0*x*+35927.1	0.9995
Chlomezanone		4.44-66.61	0.09	0.27		*y*=5611.4*x*-5526.4		0.9984		*y*=5109.9*x*+1581.5		0.9984			*y*=5388.9*x*-200.9	0.9992
7-Methoxycoumarin		4.47-67.12	0.09	0.27		*y*=10411.2*x*-1891.4		0.9970		*y*=9349.2*x*+18744.4		0.9957			*y*=9384.9*x*+11304.2	0.9962
Carbromal		15.37-230.60	0.31	0.92		*y*=5335.2*x*-2853.5		0.9993		*y*=4815.5*x*+11084.2		0.9967			*y*=5017.0*x*+4838.6	0.9997
Acrivastine		6.10-91.56	0.12	0.37		*y*=64002.7*x*+74835.0		0.9998		*y*=63540.8*x*+92476.5		0.9992			*y*=63142.6*x*+120966.0	0.9987
Erythromycin		5.12-76.75	0.10	0.31		*y*=953.5*x*-132.1		0.9992		*y*=1900.3*x*-1060.9		0.9997			*y*=1968.6*x*+584.4	0.9990
Haloperidol		5.65-84.71	0.11	0.34		*y*=12113.3*x*+7007.9		0.9993		*y*=11956.4*x*-1775.2		0.9992			*y*=12072.1*x*+17832.2	0.9984
Nalidixic acid	4.72-70.78		0.09		0.28		*y*=114282.0*x*+158470.0	0.9979		*y*=111433.0*x*+222548.0	0.9967				*y*=106571.0*x*+235781.0	0.9955
Xylometazoline	4.51-67.60		0.09		0.27		*y*=41113.4*x*-21400.5	0.9993		*y*=38636.3*x*-10143.7	0.9999				*y*=39005.2*x*+21841.1	0.9994
Diphenylpyraline	7.05-105.71		0.14		0.42		*y*=38932.7*x*+18564.1	0.9986		*y*=38944.9*x*-37850.1	0.9999				*y*=39950.7*x*-4436.5	0.9998
7-Methylcoumarin	15.56-233.36		0.31		0.93		*y*=11331.4*x*+14817.6	0.9988		*y*=10369.9*x*+74516.1	0.9956				*y*=11659.7*x*+2476.0	0.9987
Flumequine	4.71-70.58		0.09		0.28		*y*=101785.0*x*-5403.1	0.9994		*y*=101650.0*x*+142349.0	0.9978				*y*=102633.0*x*+19800.3	0.9991
Propyphenazone	5.55-83.32		0.11		0.33		*y*=66402.5*x*-9161.4	0.9994		*y*=67412.6*x*+19580.2	0.9998				*y*=63838.9*x*+61146.7	0.9976
Fluoxymesterone	5.68-85.22		0.11		0.34		*y*=7763.0*x*+11353.0	0.9989		*y*=7816.1*x*+5969.4	0.9976				*y*=7471.3*x*+12366.5	0.9978
Mifepristone	5.20-77.98		0.10		0.31		*y*=7090.3*x*+841.9	0.9993		*y*=7342.8*x*+1728.8	0.9998				*y*=6849.7*x*+6782.7	0.9995
Adrenosterone	5.40-80.96		0.11		0.32		*y*=6614.1*x*+513.5	0.9998		*y*=6337.8*x*+1689.5	0.9993				*y*=6393.6*x*+649.3	0.9998
Fexofenadine	4.67-69.99		0.09		0.28		*y*=14943.9*x*-4626.7	0.9998		*y*=14519.7*x*+5913.2	0.9996				*y*=14518.8*x*+6640.2	0.9996
Trenbolone	4.82-72.30		0.10		0.29		*y*=28289.9*x*+13253.3	0.9998		*y*=27203.6*x*+28073.8	0.9994				*y*=27708.2*x*+28165.8	0.9988
Chlorphenoxamine	5.78-86.70		0.12		0.35		*y*=33011.9*x*+13211.3	0.9983		*y*=30879.6*x*+5884.6	0.9995				*y*=32308.3*x*+15505.9	0.9998
Boldenone	4.68-70.25		0.09		0.28		*y*=21329.8*x*+4785.4	0.9992		*y*=20527.8*x*+18611.3	0.9996				*y*=20910.1*x*+14683.9	0.9998
Nandrolone	4.99-74.81		0.10		0.30		*y*=16292.4*x*+2586.5	0.9998		*y*=15448.8*x*+13140.9	0.9989				*y*=15617.8*x*+5944.4	0.9990
Androstadienedione	5.15-77.28		0.10		0.31		*y*=31777.1*x*+8254.4	0.9990		*y*=30788.0*x*+18216.5	0.9997				*y*=30545.9*x*+13699.2	0.9992
7-Ethoxy-4-methylcoumarin	4.87-73.07		0.10		0.29		*y*=43641.5*x*+15783.9	0.9988		*y*=43085.9*x*+34108.2	0.9992				*y*=40114.7*x*+33667.4	0.9975
Methandrostenolone	6.26-93.87		0.13		0.38		*y*=39037.2*x*+29478.0	0.9993		*y*=37294.2*x*+48834.3	0.9995				*y*=37613.0*x*+50277.4	0.9989
Stanozolol	5.08-76.16		0.10		0.30		*y*=23296.2*x*-13849.9	0.9976		*y*=22110.7*x*+3172.3	0.9997				*y*=24018.3*x*-33.8	0.9998
Terbinafine	4.64-69.59		0.09		0.28		*y*=197883.0*x*-83633.2	0.9991		*y*=189490.0*x*-29929.6	1.0000				*y*=182443.0*x*-26887.2	0.9987
Norethisterone	5.63-84.43		0.11		0.34		*y*=12595.8*x*-3224.5	0.9999		*y*=12619.3*x*-1615.8	0.9995				*y*=12143.2*x*+122.1	0.9995
Flugestone acetate	4.56-68.38		0.09		0.27		*y*=5428.3*x*-1096.4	0.9990		*y*=5306.4*x*-122.9	0.9997				*y*=5163.4*x*+741.7	0.9994
21-Hydroxyprogesterone	5.07-76.05		0.10		0.30		*y*=11451.4*x*-1698.6	0.9982		*y*=11308.7*x*+584.3	0.9996				*y*=11279.0*x*-3156.4	0.9996
Androstenedione	5.81-87.21		0.12		0.35		*y*=17471.6*x*-11903.0	0.9981		*y*=17552.1*x*+4557.7	0.9983				*y*=19174.3*x*-3611.7	0.9993
Ethisterone	4.38-65.63		0.09		0.26		*y*=18595.7*x*+1074.9	0.9996		*y*=18398.4*x*-2917.6	0.9990				*y*=19414.3*x*-4426.9	0.9995
Epitestosterone	4.97-74.61		0.10		0.30		*y*=17060.6*x*-4118.5	0.9998		*y*=16327.9*x*+2126.6	0.9995				*y*=16835.2*x*-3733.7	0.9995
17-*α*-Hydroxyprogesterone	5.33-79.96		0.11		0.32		*y*=6764.9*x*-6818.1	0.9988		*y*=6132.2*x*+739.5	0.9990				*y*=6182.4*x*-711.1	0.9998
Acenocoumarol	5.31-79.72		0.11		0.32		*y*=15551.8*x*-4351.5	0.9991		*y*=13520.4*x*-617.9	0.9997				*y*=14995.2*x*-4636.0	0.9990
Drospirenone	5.51-82.69		0.11		0.33		*y*=4046.4*x*+3478.4	0.9975		*y*=4249.1*x*+307.1	0.9998				*y*=3911.0*x*+3525.2	0.9988
Altrenogest	5.19-77.92		0.10		0.31		*y*=36692.0*x*+2373.5	0.9998		*y*=34430.8*x*+37710.6	0.9993				*y*=35424.1*x*+21496.8	0.9992
Megestrol	4.82-72.35		0.10		0.29		*y*=12112.8*x*+1349.7	0.9998		*y*=11814.8*x*+8455.0	0.9996				*y*=12187.4*x*+6639.6	0.9989
Clostebol	5.25-78.70		0.10		0.31		*y*=18883.3*x*-8755.0	0.9965		*y*=17434.3*x*+14585.8	0.9993				*y*=18014.5*x*+4686.8	0.9974
Medroxyprogesterone	5.07-76.11		0.10		0.30		*y*=20956.2*x*-9269.3	0.9990		*y*=19979.6*x*+124.1	0.9998				*y*=21791.8*x*-9387.7	0.9984
Dydrogesterone	4.92-73.87		0.10		0.30		*y*=29437.4*x*-13658.7	0.9988		*y*=29802.5*x*+8945.5	0.9992				*y*=27343.9*x*+16116.6	0.9990
17-*α*-Hydroxyprogesterone 17-acetate	5.44-81.67		0.11		0.33		*y*=32104.7*x*-11354.8	0.9989		*y*=29315.2*x*+22532.3	0.9994				*y*=29553.4*x*+12438.3	0.9993
Ebastine	6.35-95.30		0.13		0.38		*y*=95695.6*x*-5086.0	0.9995		*y*=91955.3*x*+20452.1	0.9995				*y*=93525.4*x*+100308.0	0.9992
Trenbolone-acetate	5.42-81.37		0.11		0.33		*y*=100624.0*x*+65911.6	0.9977		*y*=96363.9*x*-116855.0	0.9986				*y*=99984.7*x*+71205.8	0.9988
Phenylbutazone	16.71-250.60		0.33		1.00		*y*=5567.8*x*-22493.3	0.9997		*y*=3986.4*x*-30865.2	0.9961				*y*=5291.1*x*-6485.3	0.9969
Isoimperatorin	13.40-200.97		0.27		0.80		*y*=51475.3*x*+454144.0	0.9986		*y*=51420.2*x*+439348.0	0.9962				*y*=52292.5*x*+380312.0	0.9981
Norethisterone acetate	5.60-84.05		0.11		0.34		*y*=14108.6*x*-10407.8	0.9966		*y*=12968.4*x*+4816.3	0.9999				*y*=13293.7*x*-2467.3	0.9999
Danazol	6.62-99.32		0.13		0.40		*y*=12354.0*x*+8239.5	0.9990		*y*=11759.6*x*+5276.7	0.9992				*y*=11555.5*x*+12133.3	0.9988
Pyranocumarin	4.24-63.56		0.08		0.25		*y*=136375.0*x*+208747.0	0.9988		*y*=124269.0*x*+215334.0	0.9970				*y*=140515.0*x*+169625.0	0.9997
Testosterone-17-acetate	5.33-79.93		0.11		0.32		*y*=9767.8*x*-9500.0	0.9992		*y*=9348.0*x*-3651.2	0.9995				*y*=9229.6*x*+4154.6	0.9996
19-Nortestosterone 17-propionate	4.88-73.14		0.10		0.29		*y*=21178.0*x*-19268.7	0.9994		*y*=17754.7*x*+7548.0	0.9995				*y*=22237.4*x*-3757.6	0.9997
Clostebol acetate	4.70-70.57		0.09		0.28		*y*=7939.0*x*-205.9	0.9995		*y*=6830.8*x*+1177.4	0.9985				*y*=8361.2*x*-2815.2	0.9958
Testosterone propionate	5.26-78.96		0.11		0.32		*y*=9055.7*x*-11374.1	0.9981		*y*=7838.8*x*+1484.2	0.9999				*y*=10164.9*x*-1759.9	0.9999
Nandrolone phenylpropionate	5.80-86.96		0.12		0.35		*y*=21438.1*x*-13267.2	0.9990		*y*=15349.5*x*+14977.1	0.9988				*y*=21821.7*x*+2993.3	0.9994
Atranol	19.23-288.41		0.38		1.15		*y*=4617.4*x*+38620.6	0.9986		*y*=4623.1*x*+58215.1	0.9978				*y*=4338.6*x*+53171.1	0.9972
Chlorzoxazone	15.24-228.60		0.30		0.91		*y*=1300.0*x*+4343.8	0.9993		*y*=1286.2*x*+8364.5	0.9983				*y*=1292.3*x*+6615.1	0.9988
Chloroatranol	4.80-72.02		0.10		0.29		*y*=6564.3*x*+714.3	0.9995		*y*=6277.3*x*+4689.4	0.9995				*y*=6182.5*x*+3986.7	0.9986
Dienestrol	14.81-222.13		0.30		0.89		*y*=299.9*x*-1737.4	0.9962		*y*=262.5*x*+480.6	0.9994				*y*=284.4*x*-114.7	0.9998
Hexestrol	16.25-243.81		0.33		0.98		*y*=70.6*x*-210.1	0.9976		*y*=64.1*x*-119.4	0.9992				*y*=72.7*x*-463.7	0.9983

### 2.5 回收率和精密度

在水基类、乳液膏霜类和油基类的空白样品中分别添加低、中、高(1倍LOQ、2倍LOQ、10倍LOQ)3个水平的87种禁用原料混合标准溶液,根据所建立的方法进行加标回收试验,每个加标水平重复测定6次,并计算回收率和相对标准偏差。结果如表3所示,水基类、乳液膏霜类和油基类样品中87种禁用原料的回收率分别为84.8%~115.4%、82.0%~114.9%和81.7%~112.6%,相对标准偏差分别为0.4%~9.1%、0.4%~9.1%和0.6%~9.9%。以上结果表明,所建立的化妆品中87种禁用原料的高通量检测方法准确、可靠,可用于实际样品的分析。

**表3:** 87种禁用原料在3种化妆品基质中的回收率和相对标准偏差(*n*=6)

Compound	Toner matrix									Cream matrix									Oil matrix							
	LOQ			2 LOQ			10 LOQ			LOQ			2 LOQ			10 LOQ			LOQ			2 LOQ			10 LOQ	
Rec./%	RSD/%	Rec./%	RSD/%	Rec./%	RSD/%		Rec./%	RSD/%	Rec./%	RSD/%	Rec./%	RSD/%		Rec./%	RSD/%		Rec./%	RSD/%		Rec./%	RSD/%
Ranitidine	108.4	4.6		97.6	3.8		96.7	4.5		106.1	6.5		96.3	4.6		99.9	3.9		102.2	6.3		93.1	5.2		99.0	1.6	
Sulfacetamide	95.1	3.8		91.4	2.6		84.8	2.7		88.0	4.4		88.0	1.3		88.5	1.6		92.3	3.0		97.3	4.2		95.4	0.6	
Dimetridazole	105.8	3.2		95.6	3.8		87.4	2.8		92.2	3.3		90.4	3.8		91.4	4.1		101.1	2.6		95.4	4.1		99.1	2.3	
Ronidazole	104.5	2.3		99.0	3.8		91.8	1.8		92.9	2.5		98.1	3.9		94.8	0.9		90.2	4.1		98.0	3.2		99.6	1.8	
Sulfadiazine	101.6	2.6		107.4	7.4		99.7	2.5		96.1	4.7		107.0	2.3		104.0	1.0		85.9	3.0		101.6	4.6		102.9	2.4	
Emedastine	101.2	5.1		111.6	2.5		104.5	3.1		87.1	5.6		91.9	6.9		104.3	4.0		104.7	7.8		109.5	4.0		98.0	7.6	
Doxylamine	101.0	5.2		106.4	3.9		101.4	0.8		102.0	7.2		95.7	5.0		103.0	2.0		94.1	6.1		102.9	3.2		103.7	4.0	
Trimethoprim	93.0	3.9		100.7	2.7		97.3	3.4		87.7	3.9		98.0	2.2		98.0	3.1		88.9	5.0		99.0	2.0		103.8	2.5	
Marbofloxacin	105.7	7.0		111.9	2.0		98.0	4.8		91.7	8.4		98.0	6.5		109.8	3.9		84.2	4.9		112.6	8.5		95.4	5.6	
Tinidazole	90.2	6.1		106.8	4.5		99.4	2.7		89.1	7.5		108.7	2.8		99.2	4.0		92.0	5.8		103.1	2.9		100.2	1.6	
Lomefloxacin	93.7	4.8		97.7	5.5		93.3	1.2		89.8	7.5		94.1	5.2		98.6	2.6		95.0	5.5		99.2	2.2		97.4	2.5	
Danofloxacin	92.1	4.4		112.0	5.4		97.3	3.8		105.4	4.6		102.8	6.3		113.5	3.2		92.5	5.2		110.0	4.4		97.6	8.8	
Methoxyphenamine	91.9	3.8		97.0	2.8		96.0	1.5		98.2	5.4		100.7	3.1		96.7	2.4		86.9	5.3		99.3	1.9		105.6	1.6	
Sulfamethazine	104.0	6.6		115.4	4.1		107.7	3.3		94.3	9.1		113.4	4.3		105.6	2.2		89.6	4.1		103.4	5.2		102.9	3.0	
Orbifloxacin	96.0	5.1		97.4	2.6		95.8	1.9		88.6	3.6		89.8	3.7		91.8	2.4		95.2	4.9		98.6	4.2		98.7	1.0	
Naphazoline	101.7	2.4		101.2	4.3		95.8	2.6		98.7	3.5		103.1	3.7		99.2	2.5		86.1	4.5		98.4	3.9		102.7	3.1	
Ornidazole	100.3	6.2		97.5	4.6		93.8	2.4		94.1	3.6		95.8	3.0		94.1	1.9		102.8	2.7		98.0	3.4		94.7	2.2	
Gatifloxacin	101.8	6.8		98.0	4.5		97.6	1.6		99.1	5.3		98.3	3.5		98.7	3.0		92.2	5.6		96.7	4.1		96.7	2.9	
Sparfloxacin	94.1	8.5		94.6	3.8		88.8	5.8		82.0	7.3		86.0	5.9		91.7	6.3		105.8	3.2		101.6	6.6		95.9	4.4	
Methapyrilene	98.9	2.6		98.9	2.8		99.0	1.6		101.0	3.9		99.9	5.5		101.3	2.2		97.9	3.3		102.1	2.0		105.8	2.2	
Cinoxacin	93.2	4.1		99.0	7.1		98.9	5.6		96.0	3.8		93.7	3.3		91.7	1.4		94.8	2.1		97.8	6.8		95.5	1.1	
Triprolidine	112.2	4.1		109.5	6.1		103.8	2.8		102.8	3.1		100.2	6.9		99.2	1.7		90.5	8.2		99.6	4.6		107.9	3.0	
Bromisoval	102.3	4.6		95.1	5.6		94.1	2.9		86.3	3.9		92.1	3.5		98.6	4.7		98.8	5.3		94.6	4.3		99.3	1.5	
Xanthotol	93.5	5.9		96.1	5.1		96.6	1.9		85.3	4.9		99.0	4.5		100.3	5.6		103.5	6.1		101.5	3.0		100.6	3.0	
Ketotifen	93.1	3.3		100.7	2.3		98.4	1.7		91.4	3.0		100.4	3.0		101.0	3.2		89.6	1.8		101.5	2.8		102.8	2.0	
Phenacetin	94.2	5.9		107.3	1.0		96.4	1.1		90.1	2.3		99.3	3.8		94.5	1.9		90.9	2.4		102.8	2.7		98.9	2.2	
Oxolinic	93.9	2.6		100.7	3.8		96.3	3.2		85.2	5.2		98.7	6.8		101.7	2.5		94.9	3.2		104.1	4.5		101.2	4.4	
Mizolastine	97.0	2.5		102.5	3.6		98.6	2.4		86.4	4.6		103.1	6.6		99.4	2.6		88.4	4.2		104.7	3.2		103.1	4.8	
Olopatadine	103.3	1.7		98.8	4.3		92.6	5.0		90.8	2.2		93.2	5.9		95.0	5.7		101.1	5.7		99.8	3.6		98.0	4.2	
Decloxizine	99.6	4.2		102.8	2.5		99.8	1.1		94.6	1.8		102.9	1.7		103.4	1.7		95.0	2.2		98.8	2.4		103.0	2.1	
Chlomezanone	111.3	2.8		102.8	3.2		96.1	3.9		103.3	2.2		101.6	4.3		101.4	2.6		99.4	6.8		90.8	8.4		103.2	2.0	
7-Methoxycoumarin	107.8	6.1		101.7	5.9		92.8	4.7		95.7	5.3		93.8	4.5		91.3	3.0		88.3	6.8		94.4	6.1		98.1	3.0	
Carbromal	103.1	5.0		99.4	2.8		96.5	1.8		88.3	5.8		94.5	0.8		98.2	2.0		101.9	3.3		103.6	2.2		105.6	3.2	
Acrivastine	97.4	3.5		103.9	2.1		98.1	2.1		89.7	3.6		103.3	1.5		100.8	1.6		86.2	2.1		99.9	2.8		98.3	1.4	
Erythromycin	112.7	5.0		112.3	4.6		113.4	2.6		104.2	5.4		113.9	5.0		114.4	2.9		105.9	6.2		111.4	4.7		104.8	3.2	
Haloperidol	100.0	3.4		107.3	3.5		100.3	1.1		93.0	5.0		101.6	5.1		102.4	4.2		87.7	4.6		99.0	4.6		104.1	2.2	
Nalidixic acid	95.6	5.6		102.7	4.0		97.5	3.3		92.6	6.0		106.6	2.3		102.2	3.6		92.2	2.4		104.0	5.4		101.9	4.2	
Xylometazoline	108.8	3.2		102.2	3.3		97.6	2.7		97.3	2.2		98.6	3.7		98.9	1.6		90.9	5.0		98.5	2.3		106.1	2.4	
Diphenylpyraline	96.0	1.3		99.9	3.2		97.9	1.9		96.4	3.9		100.1	2.4		100.0	3.8		99.5	5.0		100.9	2.6		100.7	3.3	
7-Methylcoumarin	107.2	7.0		104.3	4.7		98.4	7.2		95.0	3.9		97.8	2.4		91.6	5.0		104.0	3.6		102.0	6.4		99.9	4.1	
Flumequine	104.4	3.8		100.8	3.3		99.1	3.5		93.2	3.9		98.8	2.1		98.1	2.6		98.6	1.7		99.2	5.7		95.2	1.4	
Propyphenazone	104.3	2.5		106.3	3.7		101.0	2.7		95.6	2.8		99.7	1.4		101.7	2.3		90.2	2.2		100.7	3.3		109.1	2.7	
Fluoxymesterone	92.4	6.9		99.6	3.2		97.8	1.7		88.8	3.6		98.7	2.9		98.0	2.6		89.7	4.1		102.7	3.1		102.0	4.0	
Mifepristone	105.9	3.8		108.5	3.0		101.5	5.2		92.1	4.3		102.7	5.6		102.5	4.7		95.5	5.9		106.4	5.5		108.9	2.0	
Adrenosterone	94.4	3.9		96.1	1.7		95.9	3.9		93.7	3.7		94.4	5.5		98.8	3.5		97.6	4.8		96.3	4.0		102.1	2.8	
Fexofenadine	107.6	5.1		104.7	3.3		100.0	2.2		92.9	3.7		94.2	4.5		100.6	2.4		90.2	4.3		97.3	3.6		105.4	2.4	
Trenbolone	100.4	5.9		104.2	2.1		96.1	2.6		92.4	3.7		100.7	3.9		102.3	1.8		94.8	2.1		100.0	2.1		101.4	3.8	
Chlorphenoxamine	96.0	3.1		98.7	3.0		96.8	2.7		92.2	3.2		94.1	2.6		94.3	2.1		94.7	4.2		98.8	1.1		101.9	1.9	
Boldenone	104.5	2.3		104.4	4.0		97.5	2.0		89.6	4.1		99.8	2.4		98.7	1.2		94.9	3.4		99.8	3.6		101.2	2.0	
Nandrolone	93.5	7.0		99.1	5.3		94.9	2.9		90.5	3.2		94.9	2.2		96.7	3.0		97.8	3.5		98.8	3.2		101.4	2.8	
Androstadienedione	98.0	2.8		100.8	1.7		99.0	1.8		93.5	2.4		96.7	2.4		97.9	2.2		96.6	3.2		98.9	2.8		104.2	3.0	
7-Ethoxy-4-methylcoumarin	100.9	5.5		102.8	4.9		95.9	3.0		90.3	4.8		98.1	2.3		95.3	2.4		93.0	5.4		101.2	5.0		103.7	3.1	
Methandrostenolone	100.8	2.3		102.9	3.4		97.6	2.1		90.8	2.2		99.5	2.3		101.0	1.4		92.7	1.3		98.7	3.8		103.0	2.1	
Stanozolol	107.0	3.6		105.7	3.9		103.0	3.3		91.3	4.7		98.5	4.8		99.4	2.4		99.7	2.3		100.7	3.9		103.7	3.0	
Terbinafine	104.7	3.0		105.2	2.3		101.3	1.7		90.9	2.6		95.4	3.5		101.4	2.7		81.7	2.3		91.3	1.6		104.6	0.7	
Norethisterone	99.8	4.1		98.5	3.5		96.2	2.8		95.1	2.6		95.4	2.6		95.2	1.4		98.9	1.5		99.3	2.7		100.6	2.9	
Flugestone acetate	98.7	4.7		95.8	3.7		94.5	3.2		93.8	3.0		93.9	3.1		95.8	2.0		96.6	6.3		95.6	4.5		99.1	2.9	
21-Hydroxyprogesterone	99.1	2.7		99.8	5.2		98.2	3.4		89.2	6.1		92.4	4.2		94.9	3.6		103.1	2.1		99.1	3.7		103.9	1.0	
Androstenedione	110.9	2.9		106.9	2.9		104.8	1.4		92.9	3.5		96.1	3.3		100.3	3.4		101.9	3.7		101.0	4.5		105.1	4.3	
Ethisterone	101.4	9.1		104.1	4.4		101.2	4.7		87.7	4.3		91.5	4.4		97.1	1.7		102.4	4.5		100.9	4.2		103.4	3.8	
Epitestosterone	104.0	0.4		100.2	3.9		99.7	2.5		92.5	6.0		96.5	2.2		100.4	1.4		103.2	3.3		102.5	3.4		102.1	3.2	
17-*α*-Hydroxyprogesterone	109.0	2.8		100.8	4.7		93.1	2.8		90.9	5.6		96.7	3.6		98.6	4.2		101.2	4.1		94.3	4.9		100.7	2.9	
Acenocoumarol	106.7	3.6		100.8	7.8		97.3	3.0		96.7	6.8		96.7	5.5		104.2	6.7		98.6	6.1		96.8	4.8		98.1	5.0	
Drospirenone	95.3	1.8		100.4	3.5		101.2	4.3		87.4	4.1		94.4	4.0		100.1	2.3		86.7	4.3		99.6	6.4		106.8	2.2	
Altrenogest	102.3	2.2		102.5	1.9		96.7	3.3		95.3	3.0		100.5	3.1		97.8	3.2		93.0	4.7		99.8	4.4		100.0	2.7	
Megestrol	105.1	6.9		102.1	3.6		98.1	2.3		104.3	2.7		99.8	4.2		97.0	3.9		91.8	6.7		95.9	4.2		100.0	1.3	
Clostebol	105.5	3.0		96.7	6.2		98.7	5.7		87.6	7.5		89.2	4.5		96.7	3.6		93.7	5.4		97.5	5.5		99.0	5.3	
Medroxyprogesterone	106.0	4.9		104.3	3.4		101.7	2.8		91.8	4.1		98.5	2.2		101.3	3.0		103.0	2.8		99.2	5.0		103.8	2.9	
Dydrogesterone	108.0	3.9		105.5	2.6		97.2	3.8		92.3	4.3		95.8	5.3		100.0	0.4		95.5	4.2		99.2	3.0		102.2	2.6	
17-*α*-Hydroxyprogesterone 17-acetate	104.0	4.7		99.7	4.4		95.4	3.1		92.2	4.2		95.0	6.6		95.3	3.8		96.9	4.0		95.8	6.8		102.8	3.3	
Ebastine	102.2	3.0		104.6	2.8		103.8	0.9		90.5	2.2		101.6	1.3		104.8	0.8		90.0	3.0		99.9	3.8		108.3	1.0	
Trenbolone-acetate	95.6	2.5		100.9	2.3		100.0	2.4		97.5	5.0		98.1	2.6		100.5	1.5		91.0	3.5		96.6	4.1		102.5	1.7	
Phenylbutazone	113.1	2.8		109.1	4.2		97.2	5.2		112.5	3.6		94.6	5.9		102.9	5.1		98.8	5.8		102.5	6.5		108.3	3.5	
Isoimperatorin	99.4	4.5		113.0	3.4		109.0	1.6		88.4	7.7		110.8	2.6		103.2	1.3		89.8	5.7		110.6	6.1		106.6	1.1	
Norethisterone acetate	109.6	4.0		102.6	3.6		97.5	2.9		97.4	3.6		100.6	2.8		101.8	2.5		100.9	4.1		101.0	1.7		101.8	3.2	
Danazol	93.7	3.5		98.2	3.5		97.0	1.5		89.3	4.2		98.3	2.7		104.2	3.8		94.4	2.9		98.7	4.8		101.8	2.5	
Pyranocumarin	89.5	4.5		103.3	2.2		104.1	1.8		90.4	2.5		99.3	2.3		103.5	2.9		90.1	3.0		101.2	2.9		103.8	1.6	
Testosterone-17-acetate	111.2	2.9		101.7	0.5		98.8	2.6		92.2	5.3		98.4	3.5		107.2	2.1		90.0	3.1		88.7	9.9		106.7	4.4	
19-Nortestosterone 17-propionate	109.2	3.6		102.8	2.6		96.3	4.2		90.6	2.3		95.8	2.5		101.4	3.8		91.3	2.2		92.0	4.2		97.4	3.0	
Clostebol acetate	99.5	6.0		105.6	5.3		105.4	7.1		98.2	8.9		104.2	3.3		109.4	4.8		104.1	6.0		100.2	7.9		108.2	4.9	
Testosterone propionate	111.2	3.7		107.4	2.8		102.6	2.9		89.0	7.9		97.9	4.2		103.7	2.4		85.7	4.5		87.7	5.5		98.2	5.1	
Nandrolone phenylpropionate	102.5	5.7		105.6	3.1		102.2	3.2		85.4	5.4		100.7	2.1		114.9	2.4		86.8	5.2		92.7	4.4		102.3	5.7	
Atranol	99.2	3.4		110.6	3.0		99.3	0.7		91.9	1.8		110.3	2.6		101.7	2.6		84.2	4.3		106.6	3.0		102.7	1.6	
Chlorzoxazone	104.9	6.3		106.4	2.6		97.4	0.8		86.4	8.2		102.6	5.1		99.6	2.6		96.6	4.8		104.7	2.4		101.7	2.2	
Chloroatranol	104.2	7.7		101.9	3.1		99.1	1.4		86.5	7.6		97.6	3.2		102.0	2.2		94.4	6.1		99.6	7.0		102.6	2.1	
Dienestrol	104.1	7.6		105.9	8.5		87.4	9.0		108.1	5.7		104.2	3.0		111.7	4.1		91.5	5.0		94.8	7.7		97.3	2.5	
Hexestrol	87.3	9.1		91.8	8.4		86.6	3.0		102.6	6.9		105.8	8.2		103.8	6.8		108.2	8.9		97.4	7.9		90.2	2.8	

### 2.6 实际样品测定

采用所建立的方法对日常监督抽检中的349批化妆品进行分析,在8批样品中检出了禁用原料。其中,有2批样品检出甲氧苄啶,检出含量分别为53.6 mg/g和15.0 mg/g;此外,1批样品检出特比萘芬,1批样品检出萘甲唑啉,3批样品检出7-甲氧基香豆素,1批样品检出7-甲基香豆素,检出含量均大于LOD、小于LOQ。实验结果表明,非法添加《化妆品安全技术规范》(2015年版)标准方法以外的禁用原料的现象已经较为普遍。因此,本方法的建立有利于加强化妆品安全监管,为化妆品的风险监测和高通量筛查提供技术支撑。

## 3 结论

本研究建立了基于超高效液相色谱-串联质谱测定化妆品中8类共87种禁用原料的方法。所建立的方法操作简单、高效,定性、定量准确,适用于水基类、乳液膏霜类和油基类3类常见化妆品基质的测定。本研究在进一步规范化妆品市场、服务化妆品安全监管等方面具有较强的实用价值。
